# Protocol for applying an enhanced quality-by-design program across the translational science spectrum

**DOI:** 10.1017/cts.2025.10200

**Published:** 2025-11-12

**Authors:** Allison Zumberge Orechwa, Leslie Aguilar, Megan Castiel, Cathelin Huang, Jeanne Dzekov, Nicole M.G. Maccalla, Wendy Mack, Zoe Mele, Kaelyn Moses, Cecilia Patino-Sutton, Saira Shah, Amytis Towfighi, Thomas Buchanan

**Affiliations:** 1 Southern California Clinical and Translational Science Institute, University of Southern California, Los Angeles, CA, USA; 2 Rossier School of Education, University of Southern California, Los Angeles, CA, USA; 3 Department of Population and Public Health Sciences, Keck School of Medicine, University of Southern California, Los Angeles, CA, USA; 4 Department of Neurology, Keck School of Medicine, University of Southern California, Los Angeles, CA, USA; 5 Department of Medicine, Keck School of Medicine, University of Southern California, Los Angeles, CA, USA

**Keywords:** Translational science, quality by design (QbD), clinical research efficiency, operations, risk-based monitoring

## Abstract

The quality by design (QbD) framework holds promise for improving success rates for completion of clinical studies, which often fail to complete on time. Initially used in manufacturing, the framework is now frequently applied to clinical trials to anticipate risks and avoid challenges that impact study completion or the credibility of results. The Southern California Clinical and Translational Science Institute created and implemented a program based on QbD to increase the success of research studies, including clinical trials and other study designs, being conducted by scholars in our Mentored Career Development Program and awardees in our Pilot Grant Programs. The program’s three components are QbD Design Studios, project management, and team science support. The overall goal is to increase study quality and efficiency, thereby improving study completion success rates and, ultimately, driving innovations in healthcare and public health. The current article describes QbD program elements in detail, along with preliminary results from initial implementation, approaches for evaluating the program’s implementation and impact on study success, and plans to disseminate the program widely.

## Introduction

While resources are available to clinical and translational researchers to aid in rigorous study design and conduct, structured pre-study approaches to anticipating and addressing logistical issues that may hamper study conduct remain insufficient. Unanticipated problems, such as inadequate staffing [1], inefficient recruitment strategies [2], or unavailable trial-related supplies [3], may only become evident to the researcher after their study is already underway. The consequences of unresolved issues range from minor study delays to study termination, which may equate to wasted time and money, unanswered research questions, and failure of investments by study participants and sponsors to have meaningful real-world impact. Proactive and thoughtful planning prior to a study’s launch could help a research team avoid or mitigate disruptions and ensure successful research outcomes.

The National Center for Advancing Translational Science (NCATS) is invested in supporting the development of innovations that overcome long-standing challenges along the translational research pipeline. One promising framework for mitigating risks to study completion is quality by design (QbD), a systematic approach to reduce errors and maximize quality through ongoing learning and continuous improvement throughout a study’s lifecycle [4]. Historically used in the manufacturing industry to reduce production waste, the QbD framework has more recently been applied to clinical research, where quality is defined as the absence of errors that matter – that is, errors that impact the safety of trial participants or the credibility of trial results. Poor quality research is both potentially hazardous to trial participants and also unlikely to translate to improvements in patient health.

QbD methods proactively build quality into the scientific and operational design of studies. The intent of QbD is to facilitate early identification of study risks during study development and preparation. The QbD approach involves cross-functional discussions between the research team and stakeholders to rigorously evaluate study plans from multiple perspectives. The shared goal is to identify critical drivers of quality and important risks to quality, and then determine approaches to avoid problems or correct them as soon as they arise. This proactive approach prepares the research team to periodically review the critical-to-quality factors and evaluate their risk control mechanisms during study conduct. After the study is concluded, teams are encouraged to capture and share lessons learned with other researchers in the organization and incorporate learnings into future studies.

The QbD framework has been adopted or referenced by organizations across the clinical research spectrum, including the FDA, Pfizer, PCORnet, the Medical Device Innovation Consortium, and CTSA hubs at Duke University and the University of California, Irvine. The Clinical Trials Transformation Initiative (CTTI), a public-private partnership established in 2007 by the FDA and Duke University, is driving the adoption of QbD through free training and resources such as checklists and guidelines for implementing QbD locally. Noting that there are many ways to adopt QbD thinking, CTTI recommends that organizations allow for flexibility in selecting specific QbD strategies to implement, given inherent differences among studies and organizations. Research to date indicates that QbD can be feasibly implemented in a variety of settings, is perceived by investigators as valuable, and can lead to improvements in study plans [5,6].

QbD differs from other quality frameworks, such as Total Quality Management (TQM), which involves long-term, continuous improvement, aiming to foster a culture centered on quality, enhance employee morale, and boost customer satisfaction [7]. TQM’s broad, organization-wide focus and emphasis on cultural transformation contrast with QbD’s more targeted, scientific, and risk-based approach, which is more closely aligned with NIH-funded Clinical and Translational Science projects. QbD proactively embeds quality into process and product design from the outset, relying heavily on data, risk assessment, and regulatory alignment. TQM seeks to instill a culture of continuous improvement throughout the organization. While QbD may deliver more immediate, measurable improvements in process robustness and regulatory compliance, especially where scientific rigor is essential, TQM’s strengths lie in its ability to drive sustained, organization-wide quality improvements over the long term but with potentially slower and less quantifiable early returns. These differences make the two approaches potentially complementary. If resources are limited, the choice between TQM and QbD ultimately depends on organizational goals, regulatory context, and readiness for cultural change.

Inspired by the CTTI QbD initiative and colleagues at the University of California, Irvine, our institute, the Southern California Clinical and Translational Science Institute (SC CTSI), developed a program around QbD principles to support our mission to advance clinical and translational science. Our approach involves hands-on support led by trained SC CTSI personnel to launch and oversee individual studies through: (1) Design Studios where diverse advisors provide tailored advice to the investigator using the QbD framework; (2) project management implemented through monthly check-ins to track progress against milestones and help resolve study impediments; and (3) team science support to ensure smooth and collaborative study conduct. The first element of our approach replicates other QbD programs; the second and third elements are enhancements that we predict will further support successful study conduct and completion. The initial focus of our program is SC CTSI-funded studies, which have historically had variable success in meeting enrollment targets, completing on time, producing publications, and garnering follow-on funding.

The objective of this article is to provide details of all QbD program elements, as well as the ongoing, mixed-methods program evaluation designed to illuminate feasibility, perceived value, barriers and facilitators, and ultimate impact on study success. We also describe our initial observations about the program’s implementation, value to study teams, and preliminary impact data. Finally, we present plans to disseminate the program widely to other researchers and organizations for broader impact on the research enterprise.

## Materials and methods

### Program development

Development of our QbD program began in 2019 with introductory training on the QbD framework and process. A QbD expert from the CTTI hosted a two-hour virtual training workshop for all faculty members and managing staff from SC CTSI. They presented the origins of the framework, case studies, and CTTI website resources.

We then assembled a team to develop program goals and plans. The overarching goal was “to implement the Quality by Design framework, enhanced by team science and project management, to increase quality and efficiency in the conduct of clinical studies.” The program focuses on projects receiving funding from the SC CTSI (see Funding Mechanisms below). Specific goals are to (a) achieve at least 80% of studies completing on time (i.e., completing study procedures by end of funding period; prior to QbD launch, on-time completion rate for SC CTSI pilot projects was 58%) and (b) expand access to QbD methodology by sharing our QbD approach with at least 20 other studies at five other sites through consultation and provision of tools and additional implementation resources. Milestones for developing the SC CTSI QbD program include launching the program, evaluating the program, adapting resources for non-trial studies, and disseminating a toolkit to other CTSA hubs and health research organizations.

We provide direct QbD support to participating studies that receive direct funding through one of three SC CTSI funding mechanisms:

- Pilot Grants ($50k for 1–2 years) that provide funds for the development of new clinical and community-based research projects
- Research projects of varying size conducted over three years by Scholars participating in our Mentored Career Development (MCD/KL2) program in clinical and translational science
- Health System Innovation Awards ($125k over 2 years) focused on improving healthcare delivery within partner health systems

While studies funded under these mechanisms all fall within the broad field of clinical and translational research, they vary widely in their disease areas and research designs. These factors will be considered in our assessment of the program as a generalizable solution. Studies are led by principal investigators (PI) from disciplines across the University of Southern California and Children’s Hospital Los Angeles. Each project underwent a peer review process and was selected as meritorious.

Once QbD program goals were defined, we collected CTTI QbD resources and created initial versions of additional guidance documents including meeting agendas, attendee lists, study project plan templates, and standard operating procedures. Current versions of these resources can be found at: https://sc-ctsi.org/resources/quality-by-design.

### Program management

Working closely with the program leaders is the QbD Specialist, who manages the QbD process for individual studies and tracks implementation data and study outcomes. The Specialist serves as the primary contact for participating PIs. Skills central to the role are project management, meeting facilitation, customer service, and familiarity with a wide range of study designs and support resources.

Program leaders created a thorough training program for QbD Specialists that includes meeting with SC CTSI directors for a formal overview of the program goals and components; exploring the CTTI and SC CTSI QbD toolkits; reviewing notes from past SC CTSI QbD Design Studios; and observing at least three QbD Design Studios led by a current QbD Specialist to understand the structure, facilitation techniques, note-taking skills, and key discussion points. Additionally, it is recommended that the Specialist completes training in project management skills and tools. A critical component of the training involves engagement with key CTSI personnel, such as pilot award administrators, to understand their roles and resources, enabling the integration of their unique expertise with diverse study needs.

The partial salary of the QbD Specialist is the QbD program’s primary operational cost. At our institution, this early career staff member devotes 0.25 FTE to the program and handles other grant management and evaluation tasks. This approach minimizes dedicated personnel costs. Further contributing to its cost-efficiency, the program requires no specialized software or infrastructure, instead leveraging readily available institutional tools for project management and virtual meetings. Additionally, expert advisors contribute roughly two hours of their time per study, with an average of eight advisors per design studio. As these advisors are primarily faculty or staff internal to SC CTSI, their contributions are considered in-kind support, not incurring direct hourly fees. Honoraria are available for patient representatives or other external advisors as needed.

### QbD process for participating studies

#### QbD introduction

**Figure 1.**
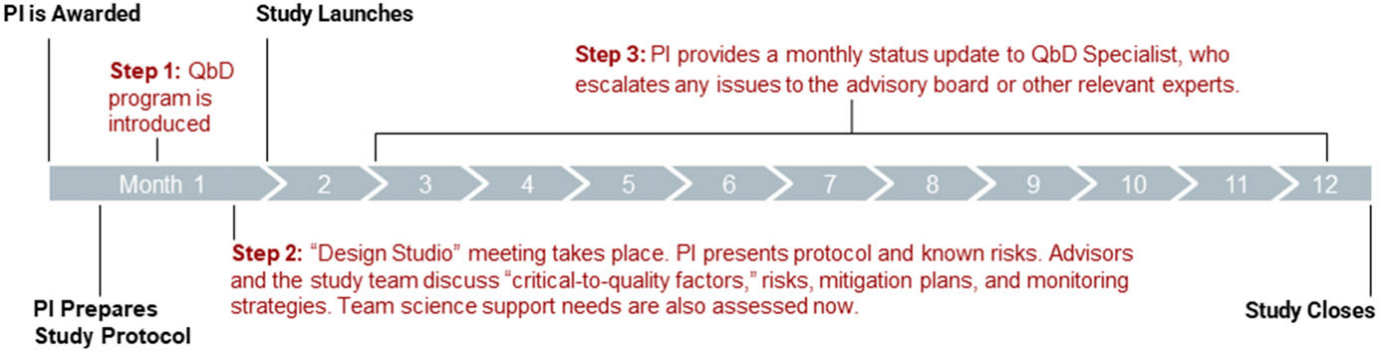
QbD timeline: Typical timeline of quality-by-design program steps relative to a 12-month pilot study. PI = principal investigator.

Figure 1 depicts the QbD process for individual studies. Within two weeks of award notification, the QbD Specialist emails the participating study PI with a detailed program description and schedules a QbD Introduction Meeting. At the QbD Introduction Meeting, the QbD Specialist provides an overview of the QbD framework, describes the QbD process, and discusses the importance of a proactive and multidisciplinary approach. The Introduction Meeting also allows the Specialist to learn more about the study objectives, design, and plans and, in collaboration with the study team, assemble relevant subject matter experts who will advise the study team about potential logistical issues during the upcoming Design Studio. Additionally, the PI can ask questions and gain further insights into how QbD may enhance their study conduct and rigor.

In many cases, the Introduction Meeting occurs before the PI has prepared a detailed study protocol. A protocol is necessary because it allows advisors to thoroughly evaluate the study plan, identify potential roadblocks, and provide valuable insights for smooth study conduct. In such cases, the QbD Specialist shares protocol templates from the National Institutes of Health and offers feedback on protocol drafts [8]. In all cases, the study PI provides a draft of the detailed protocol at least one week before the Design Studio to allow attendees ample time for review. Protocols are typically revised following Design Studios to incorporate necessary changes.

#### Design Studio

**Figure 2.**
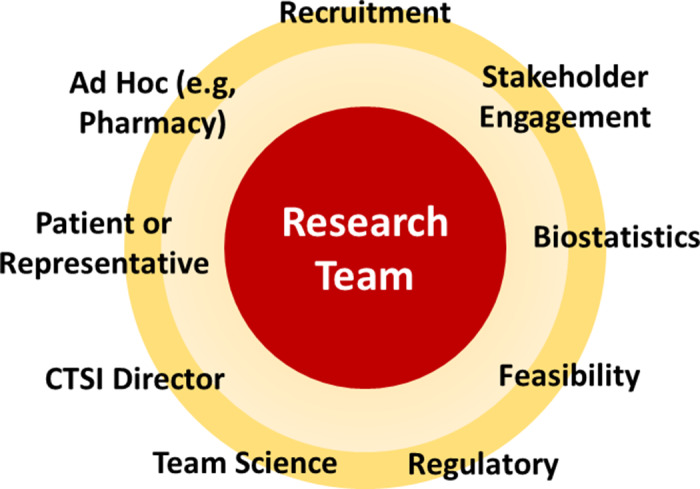
QbD Design Studio attendees: Advisors with expertise in multiple disciplines provide tailored advice to awarded research teams. CTSI = Clinical and Translational Science Institute.

Once a protocol draft is available, the QbD Specialist schedules the Design Studio, a virtual meeting between the study team and an “advisory board” of subject matter experts who provide tailored advice to the investigator using the QbD framework. Each advisory board is unique to the study needs but typically includes scientists, mentors, and other research professionals with extensive research experience and collective expertise in biostatistics, regulatory aspects, stakeholder engagement, participant recruitment, study operations, team science, and informatics (Figure 2). A representative from the study’s target population (e.g., patient, community member) is a member of the advisory team to provide perspectives on feasibility and participant engagement plans. The interdisciplinary nature of the board ensures that diverse perspectives are provided to the study team about needs and risks associated with the study, from initiation through enrollment, study conduct, data analysis, and dissemination. Three documents are provided to all advisory board participants at least a week before the Design Studio: (1) the meeting agenda, (2) the study protocol, (3) a QbD Critical to Quality (CTQ) Factors Principles Document, either the CTTI version for clinical trials [9], or a version we adapted for health services research using an implementation science framework [10]. We encourage study teams and advisors to familiarize themselves with all documents prior to the Design Studio.

The content of the Design Studio is built around the CTTI QbD CTQ Factors Principles Document. The CTTI Principles Document is designed to promote discussion rather than be used as a checklist of tasks, consisting of a table that describes 21 CTQ Factors to consider in conducting a study, for example, randomization, accrual, data monitoring and management, and collaborations. The document provides corresponding examples of issues to consider in evaluating risks, for example, “Are the inclusion/exclusion criteria clearly defined?” The questions help advisors prepare study-specific questions for the study team. It also helps the study teams prepare to answer questions from advisors during the Design Studio.

The Design Studio follows a standard, templated agenda. Following introductions, the QbD Specialist states the meeting goals: (1) identify risks to successfully completing the study, and (2) develop risk mitigation plans. Next, the PI briefly describes the study aims and protocol elements, including the target enrollment, participant recruitment plan, source(s) of data, data collection plans, analysis plans, staffing plans, and any external partners involved. The advisors then ask clarifying questions to understand the study and to assess the study team’s capabilities in team science, particularly if the team is large, interdisciplinary, and/or involves diverse stakeholders. Advisors may offer resources for team science support, such as consultations and virtual software recommendations, through the SC CTSI Team Science program. The program provides support consistent with the evidence that cross-disciplinary and boundary-spanning research teams are generally more productive and innovative but benefit from institutional resources to work efficiently and effectively as a team [11]. The PI concludes their presentation by stating any concerns regarding completion of the study (i.e., risks to Critical to Quality Factors), for example, inadequate staffing or lengthy survey instruments.

The bulk of the Design Studio time is devoted to discussion of approaches to address concerns and any additional concerns raised by advisors. If the list of risks is extensive, the QbD advisors along with the PI prioritize the top 3–5 risks to focus their discussion of mitigation strategies. Through a collaborative process facilitated by the QbD Specialist, the advisors offer the researcher practical solutions to potential issues and a list of immediate action items needed before launching the study. For example, in a Design Studio for a clinical trial comparing the efficacy of group vs. individual medical visits in neurology clinics, advisors identified a potential technology barrier for participants joining virtual group settings. They recommended helping each patient prepare the technology prior to the clinical visit. For a study examining AI-based glaucoma screening, advisors noted the urgency of establishing a referral pathway for patients flagged as having glaucoma. In a multicenter COVID-19 vaccine trial, advisors noticed a lack of tools to track participant enrollment against targets and provided a tool with monitoring algorithms.

#### Project management

Following the Design Studio, the QbD specialist circulates meeting notes, requests the study team submit a set of milestones to track progress after the study’s launch, elicits feedback from PIs on the Design Studio process, and explains the QbD Specialist’s continued involvement in a project management capacity. The QbD Specialist contacts the study team at one-month intervals to check on study progress against milestones, inquire about the risks identified in the Design Studio, and document the success of mitigation strategies recommended by the advisors. If the study team identifies challenges, the Specialist refers them to the advisors, SC CTSI, or other institutional resources to address the problem. At the study closure, the Specialist collects data on the success of the project, such as meeting enrollment targets and other milestones. Longer-term follow-up occurs annually through the CTSI-wide annual survey. Outcomes include study-related publications, follow-on funding, and real-world impact according to the Translational Science Benefits Model (TSBM) [12].

### Evaluation plan

We are assessing the implementation and impact of the QbD program in an ongoing manner over six years, covering at least two subsequent funding cycles and three-year follow-up following award completion. This will provide a sample of 30–35 studies, depending on the number of studies funded in each cycle. Outcome measures, summarized in Table 1, include the following:

**Table 1.** Primary program impact measures

Program impact measure	Evaluation method	Measurement timepoint
Perceived value to participating researchers	Interviews with individual study PIs	After Design Studio and after study completion
Publication success	Comparison to baseline through institutional surveys and PubMed monitoring	Annually for three years
Time to publication	Comparison to baseline through record review	End of evaluation period
Risks anticipated	Frequency counts of risks documented during Design Studio and identified by attendees as primary risks	After Design Studio
Barriers encountered	Frequency counts of actual barriers reported by study PIs	After study completion
Culture of quality	Questions in institutional surveys	Annually

#### Implementation evaluation

We are evaluating the degree to which participating studies received each QbD program component (introduction, Design Studio, team science support, and project management support). We also measure “quality culture” factors from the CTTI QbD Maturity Model [13], specifically the extent to which there is awareness of QbD across the SC CTSI (via a question in the institute’s annual survey), and the extent to which lessons learned are incorporated into future studies (asking PIs if they changed any study plans after reading tips and case studies posted on the QbD website).

#### Program impact

We assess satisfaction with QbD support among study PIs by asking if the QbD added value to their project, and if so, how. This “perceived value” measure serves as a primary endpoint and is assessed via individual structured interviews at two time points: immediately following Design Studios and following the close of the award. We are assessing satisfaction with each element of the program and exploring differences across characteristics such as PI seniority and study types.

We also collect quantitative metrics to help determine whether the program has met its goals of increased quality, efficiency, and on-time completion rates (80% target). Through comparisons with pilot studies previously funded by the SC CTSI, we can determine if QbD support is associated with higher publication success (as an indicator of quality) and faster time to first publication (as an indicator of efficiency). We will calculate the fraction of studies that were completed on time using Pilot Grants’ No-Cost Extension (NCE) requests as a proxy measure. Additional measures include the percentage of study milestones achieved and the time to completion of study procedures. Longer-term tracking will assess submission and award of follow-on funding proposals, as well as grant proposal scores (if available), longer-term changes in behaviors (e.g., collaborations, anticipating study risks and critical to quality factors, development of risk mitigation plans, team capacity building efforts), and real-world impacts (i.e., TSBM impact measures).

Using a concurrent mixed-methods design, we are evaluating the most common types of QbD-identified risks across all studies that received QbD support. The Principles Document provides a framework for categorizing the potential risks identified during the Design Studio that individual research teams encountered during the study. The document is also used to categorize the issues that were threats to quality that neither the study team nor the advisors had anticipated. Identification of common issues has broad implications. Common problems can reveal opportunities for improvement, institute-wide, and help direct resources toward developing generalizable solutions to common barriers, a key principle of Translational Science.

Collaborators at the University of California, Irvine, are collecting a subset of the above measures as part of their similar QbD program. This allows for a larger sample size and exploration of cross-site variation in program efficacy.

In terms of limitations of the evaluation plan, participant bias may impact data quality. During the QbD process, the participants (PIs) might overestimate their progress against milestones and downplay any issues. Similarly, they may express overly positive views of the program during post-award interviews to maintain a positive relationship with the institute. We are mitigating this potential bias throughout the evaluation process. For instance, we inform participants that their feedback will not affect their access to future institute resources. We have also tasked a team member outside the QbD team to conduct the interviews to help avoid bias. Another limitation related to data quality is the potential for minor inaccuracies in the historical 2017–2019 Pilot Grant No-Cost-Extension data stemming from incomplete records, mislabeling or errors during data entry. While every effort has been made to verify and clean the data, such errors could influence the precision of findings and should be considered when interpreting results.

### Dissemination plan

We plan to share the results of the QbD program evaluation through communication channels relevant to the translational research community, including scientific publications, presentations at the Translational Science annual conference, news articles, and a posting to the Trial Innovation Network toolbox [14]. NCATS may be interested in showcasing QbD as an exemplary Translational Science project that generates solutions to persistent logistical problems in translation.

We plan to share the program materials we have developed for participants and the online training for new QbD Specialists with other organizations. Some materials are already freely available for download on the SC CTSI website. Additional materials will include a program implementation toolkit with detailed instructions, examples, tips, and tracking tools.

## Results

We launched the QbD program in the fall of 2022. As of September 2025, we have completed Design Studios for 28 studies. Five of the 28 are completed, 20 are active, and 3 are awaiting federal or institutional review board approvals before launching.

### Design Studios

The counts provided in Table 2 indicate that implementing QbD Design Studios is feasible and the procedures are effective in identifying risks for a wide variety of study types and disease areas. Responses from post-Design Studio queries to date indicate that the program is demonstrating value to PIs. All PIs who responded to the query (26 out of 28 total, or 92.8%) answered “Yes” to the question, “Did you find the session helpful?” One PI replied, “I found the meeting very valuable. I really appreciated having a number of external perspectives to help troubleshoot my project and help me strategize about ways to circumvent some issues that may come up.”

**Table 2.** QbD program implementation to date – study types, disease areas, and risks identified

Study types (# of studies)	Prospective, retrospective, or mixed observational study (14) Health services research (9) Clinical trial (4) Formative intervention development (2)
Research topics (# of studies)	Cancer (5) Mental health (3) Glaucoma (2) Cerebral palsy (2) Interpersonal violence (1) Alzheimer’s disease (1) Vaping (1) Emergency care (1) Headaches (1) Systemic lupus erythematosus (1) Dementia (1) Stroke (1) Pediatric intestinal failure (1) Sickle cell anemia (1) Hearing loss (1) Chronic rhinosinusitis (1) Methamphetamine use disorder (1) Cerebrovascular disease (1) Pediatric liver disease (1) Hearing loss (1) Autism spectrum disorder (1)
Risks identified within CTQ factors (# of studies)	Accrual (17) Procedures supporting study endpoints and data integrity (15) Study and site feasibility (14) Dissemination (9) Eligibility criteria (7) Training (7) Data quantity (7) Collaborations (4) Data recording and reporting (4) Endpoints (4)

### Study completion

Data on NCE requests among pilot projects provide preliminary signs that QbD may be associated with faster study completion. Three (60%) of the five studies completed to date did so in the projected time period, while two (40%) received NCEs. This frequency is similar to the 42% (14 of 33) of pilot projects that received NCEs in a historical comparison group of pilot projects funded by the SC CTSI between 2017 and 2019. However, the average duration of extension (including the pilots that received 0 months of NCE) was 44% longer in the historical comparison group (4.9 vs. 3.4 months). Additional follow-up of our current cohort of studies will be required to determine if that trend will continue.

### Study progress

For exploratory analyses of study progress, we asked PIs of active studies to indicate the status of each milestone in their original project plan. Of the 20 active studies, two study PIs did not develop milestones prior to study launch, and three did not respond to our inquiries. Of the twelve studies for which we have detailed status information, eleven reached at least 50% of the milestones they expected to have reached by this point in their project timeline. One PI reported completing all expected milestones; two completed 80% or more, and eight met between 50% and 80% of their expected milestones. No projects were ahead of schedule.

### Post-study interviews

We conducted exit interviews with PIs of four of the five completed studies. Qualitative analyses of the interviews revealed that investigators described the program as a valuable experience that helped them think more deliberately about study planning and risk mitigation. Most participants viewed the introductory meeting and design studio as useful for structuring early project discussions, clarifying timelines, and identifying potential barriers. However, some noted that the benefits tapered off after the initial session due to limited follow-up or continued engagement. Investigators appreciated the opportunity to receive input from diverse reviewers and saw QbD as a constructive framework for embedding quality and proactive planning into study design. However, they differed in how much the experience changed their approach. Those newer to project management found the QbD mindset to be more transformative, while others with prior exposure saw it mainly as validation of existing practices.

Regarding delays, the most common challenges stemmed from institutional and administrative hurdles, including prolonged IRB processes, budget issues, and limited staff capacity. While a few investigators mentioned protocol amendments or recruitment adjustments that arose later in their projects, all reported completing studies on schedule and noted that any delays were due to institutional or administrative hurdles outside the scope of the QbD program. Overall, investigators endorsed QbD as an important mechanism for promoting proactive, quality-focused research planning, while recommending stronger ongoing mentorship and mid-project check-ins to sustain its value beyond the initial design phase.

Some feedback has also inspired changes to the program. For example, one PI suggested holding the Design Studio earlier in the study activation phase to better accommodate suggested changes. In response to this feedback, we started scheduling Design Studios as close to award start dates as possible while remaining flexible to the PI’s study timelines.

Results to date support the report by the University of California, Irvine, that their QbD program was feasible and valuable [5]. More detailed results on perceived value will be available after the initial round of Design Studios is complete and PI responses are analyzed in early 2026. Full program evaluation is ongoing and is expected to be completed in 2028. QbD support will continue to be provided to new study teams in parallel to program evaluation efforts.

## Discussion

The methods described above will advance our understanding of the impact of QbD on study success. The results of the rigorous, mixed-methods evaluation will inform whether and how the SC CTSI’s approach to QbD, which is rooted in principles of team science, project management, and quality management, is related to increases in study quality and timely completion.

Findings will also provide contextual information regarding how the SC CTSI QbD program is implemented, what aspects of studies it influences, and whether the framework is generalizable, particularly for studies beyond clinical trials. The detailed findings will complement the resulting toolkit of templates and guidance materials to assist others in implementing this or other QbD programs most effectively at their own institutions.

Future efforts to study QbD implementation and outcomes on a larger scale should be aimed at further refining QbD programs. For example, identifying settings and study types most conducive to QbD will allow research organizations to direct their resources to the studies that benefit the most. Similarly, identifying common issues that prevent study completion in certain study types or settings, such as transportation barriers in rural sites, would inform changes needed in research infrastructure for the benefit of multiple studies or sites. Program components that deliver the most value could help more organizations tap into the value of QbD despite resource constraints.

A primary drawback of the QbD approach that might limit widespread adoption is the added work required to implement a comprehensive program. The steps involved–introducing the program, gathering advisors, critically discussing the study plans, and amending the plans–invariably add time and energy to the overall research effort. However, as the goal of the framework is to prevent errors and streamline plans, ideally, the benefit of faster and successful completion will outweigh the initial cost. Additionally, one would expect that synchronous feedback from multiple experts is more efficient and productive than back-and-forth communication with individual advisors. Results of this evaluation will help answer the question of whether the resulting savings are worth the significant upfront investments.
